# Novel Antitumor Strategy Utilizing a Plasmid Expressing a *Mycobacterium tuberculosis* Antigen as a “Danger Signal” to Block Immune Escape of Tumor Cells

**DOI:** 10.3390/pharmaceutics7030165

**Published:** 2015-07-24

**Authors:** Yoshiyuki Koyama, Chieko Yoshihara, Tomoko Ito

**Affiliations:** 1 Japan Anti-tuberculosis Association, Shin-Yamanote Hospital, 3-6-1 Suwa-cho, Higashimurayama, Tokyo 189-0021, Japan; E-Mail: tomoko_ito@nifty.com; 2 Graduate School of Life and Environmental Sciences, Osaka Prefecture University, 1-58 Rinku-oraikita, Izumisano, Osaka 598-8531, Japan; 3 Department of Home Economics, Otsuma Women’s University, 12 Sanbancho, Chiyoda-ku, Tokyo 102-8357, Japan; E-Mail: yoshihara@otsuma.ac.jp

**Keywords:** early secretory antigenic target-6, immuno-gene therapy, non-viral, transfection, plasmid, danger signal, cytokine, *Mycobacterium tuberculosis*, immune escape, nanoparticle

## Abstract

Immune escape of tumor cells is one of the main obstacles hindering the effectiveness of cancer immunotherapy. We developed a novel strategy to block immune escape by transfecting tumor cells *in vivo* with genes of pathogenic antigens from *Mycobacterium tuberculosis* (TB). This induces presentation of the TB antigen on tumor cell surfaces, which can be recognized by antigen presenting cells (APCs) as a “danger signal” to stimulate antitumor immune response. This strategy is also expected to amplify the immune response against tumor-associated antigens, and block immune escape of the tumor. DNA/PEI/chondroitin sulfate ternary complex is a highly effective non-viral gene vector system for *in vivo* transfection. A therapeutic complex was prepared using a plasmid encoding the TB antigen, early secretory antigenic target-6 (ESAT-6). This was injected intratumorally into syngeneic tumor-bearing mice, and induced significant tumor growth suppression comparable to or higher than similar complexes expressing cytokines such as interleukin-2 (IL-2) and interleukin-12 (IL-12). Co-transfection of the cytokine-genes and the *ESAT-6*-gene enhanced the antitumor efficacy of either treatment alone. In addition, complete tumor regression was achieved with the combination of *ESAT-6* and *IL-2* genes.

## 1. Introduction

As a safe alternative to viral gene carriers, we have developed a highly effective *in vivo* gene transfection system, which consists of small plasmid complex particles with negative surface charge [1,2]. Small DNA complexes with a plasmid encoding the *granulocyte macrophage colony-stimulating factor* (*GM-CSF*) gene demonstrated efficient antitumor activity in an allogeneic B16 melanoma mouse model. Administration of the plasmid complex led to complete tumor eradication, without regrowth [1,2]. Clinical studies on primary tumor-bearing cats and dogs were also performed using the complex of plasmid encoding *feline* or *canine GM-CSF*, respectively. In some cases, apparent tumor regression was observed, but a complete response was not achieved. In those animals with established primary tumors, the tumor often escapes from immune surveillance, and transfection of cytokine genes alone was not sufficient to evoke an effective antitumor immune response.

However, several kinds of viral vectors harboring cytokines have been reported to successfully induce tumor-regression in human clinical studies [3], whereas non-viral vectors harboring the same cytokine genes showed limited therapeutic efficacy. One possible reason for the inferior efficacy of synthetic vectors compared to that of viruses is the low transfection efficiency of the non-viral systems. However, virus infection may function independently to induce tumor regression. The antitumor effect of microorganisms was initially recognized in ancient Egypt [4]. Since the pioneering work of N.G. De Pace was performed about a century ago [5], a number of clinical trials using natural [6] or genetically engineered [7] viruses to treat tumors have been conducted. The antitumor activity of the virus was primarily attributed to the cell-killing effect of the viruses proliferated in the tumor tissue. However, preclinical and clinical data suggest that in some cases virotherapy may in fact act as cancer immunotherapy [8]. Virally infected tumor cells express pathogenic viral antigens on their surfaces, which can be recognized by antigen-presenting cells (APCs) as a “danger signal” leading to activation of an immune response against the viral antigen. In addition, APCs can also enhance the immune response against simultaneously captured tumor-associated antigens.

The expression of antigenic proteins on the tumor cell surface is essential for APCs activation, and, thus, is expected to evoke an immune response against the escaping tumor cells. We speculated that similar to virus infection, transfection of tumor cells with plasmids harboring virus- or bacteria-specific antigenic protein genes could also induce pathogenic antigen presentation in the tumors. This strategy avoids the risks associated with using actual infectious organisms. Plasmid formulation would also provide the advantages of stability for storage and shipping, and cost-effectiveness.

Here, we employed the highly antigenic pathogenic proteins, *Mycobacterium tuberculosis* early secretory antigenic target-6 (ESAT-6), as an immune response-inducing antigen for the following reasons: DNAs coding antigens of *Mycobacterium tuberculosis,* such as ESAT-6, antigen 85 complex, and MPT64, were reported to induce antigen-specific responses, and can serve as a DNA vaccine against infection with tuberculosis [9]. Among them, ESAT-6 was found to have several unique properties, such as membrane-interacting [10], pore-forming [11], membrane lysing [12], and Toll-like receptor binding activities [13], which may be favorable for generating an immune-stimulating “danger signal”. Small DNA complex particles were made from plasmids encoding the *ESAT-6* gene, and the antitumor therapeutic efficacy of the pathogenic antigen transfected into syngeneic tumor-bearing mice was explored. The effect of co-transfection with cytokines such as interleukin-2 (IL-2) and interleukin-12 (IL-12) with ESAT-6 was also examined.

## 2. Experimental Section

### 2.1. Materials and Mice

Chondroitin sulfate sodium salt from shark cartilage (MW 10,000) (CS) was supplied by Seikagaku Corp. (Tokyo, Japan). Dextran (MW 180,000–220,000) was obtained from MRC Polysaccharides Co., Ltd. Polyethylenimine “Max”, (MW 40,000 in a hydrochloride salt form, comparable to MW 25,000 in a free base form) (PEI) was purchased from Polyscience, Inc. (Warrington, PA, USA). Preparation of plasmids harboring the *ESAT-6*, *murine IL-2*, or *murine IL-12* genes was carried out by Takara Bio Inc. (Shiga, Japan) by inserting these genes into pcDNA3.1 vector with a Kozak sequence (GCCACC). Amplification of these plasmids was performed by AMBiS Corporation (Okinawa, Japan). Cell culture lysis reagents and the luciferase assay substrate were purchased from Promega Corporation (Madison, WI, USA). The protein assay kit was obtained from Bio-Rad Laboratories (Hercules, CA, USA). Male C57BL/6 mice (5 weeks old) were purchased from Tokyo Laboratory Animals Science Co., Ltd. (Tokyo, Japan).

### 2.2. Cytotoxic Activity of the Plasmid Complexes

#### 2.2.1. Preparation of DNA Complex

DNA complexes were prepared in 7 mM phosphate buffer (PB) at pH 7.4 as follows: CS solution (267 μg in 600 μL) and PEI “Max” solution (132 μg in 300 μL) were added, in this order, to a solution of plasmid DNA (45 μg in 300 μL). After standing for 30 min, the mixture was diluted with condensed phosphate buffered saline to generate an isotonic solution containing a given amount of the plasmid complex.

#### 2.2.2. Evaluation of the Cytotoxicity

B16 mouse melanoma cells were seeded onto 96-well plates at 5.2 × 10^3^ cells per well, and cultured for 2 days in Eagle’s Minimum Essential Medium (EMEM) supplemented with 10% fetal bovine serum (FBS), penicillin G sodium (100 unit/mL), and streptomycin sulfate (0.1 mg/mL). The primary growth medium was then replaced with 100 μL of fresh EMEM with FBS and antibiotics. DNA complex suspensions were then added to the cells (100 μL/well), and incubated for 4 h at 37 °C. Fresh medium was added to the wells (100 μL/well), and after an additional incubation at 37 °C for 20 h, cell viability was measured by WST-1 assay.

### 2.3. Antitumor Efficacy of the Plasmid Complex

#### 2.3.1. Preparation of Plasmid Complex

The DNA/(PEI “Max”)/CS complex (1:12:8 in charge) was prepared as follows: Phosphate buffer (PB) (pH 7.4; 7.4 mM, 4720 μL), aqueous solutions of CS (593 μg in 178 μL) and PEI “Max” (294 μg in 58.7 μL) were added in that order to an aqueous solution of plasmid DNA (100 μg in 47.6 μL). After standing for 20 min, Dextran solution (50 μL; 10%) was added. The mixture was then frozen at −20 °C and freeze-dried at room temperature to give a spongy complex. It was rehydrated with 250 μL of water just before use.

#### 2.3.2. Evaluation of Antitumor Efficacy

B16 cells were cultured in minimum essential medium (MEM) supplemented with 10% fetal bovine serum (FBS), penicillin G sodium (100 unit/mL), and streptomycin sulfate (0.1 mg/mL). Male C57BL/6 mice (5 weeks) were inoculated subcutaneously with 2.0 × 10^6^ B16 cells. When the major axis of the subcutaneous tumor reached 3 mm in diameter, the DNA/(PEI “Max”)/CS ternary complex (containing 100 μg plasmid coding ESAT-6, IL-2, or IL-12) rehydrated with 250 μL water was intratumorally injected five times every other day, and the tumor size was measured every day for 125 days (*n* ≥ 4). All animal studies were carried out in accordance with the guidelines of Otsuma Women’s University (certificate numbers: 10004, 121004 and 13005).

### 2.4. ζ-Potential and Size Measurement

Plasmid DNA complexes were prepared under the same conditions as those used for *in vivo* transfection. They were diluted with water, and subjected to analysis in a particle analyzer (MALVERN Zetasizer Nano ZS, Malvern, Worcestershire, UK).

## 3. Results and Discussion

**Figure 1:**
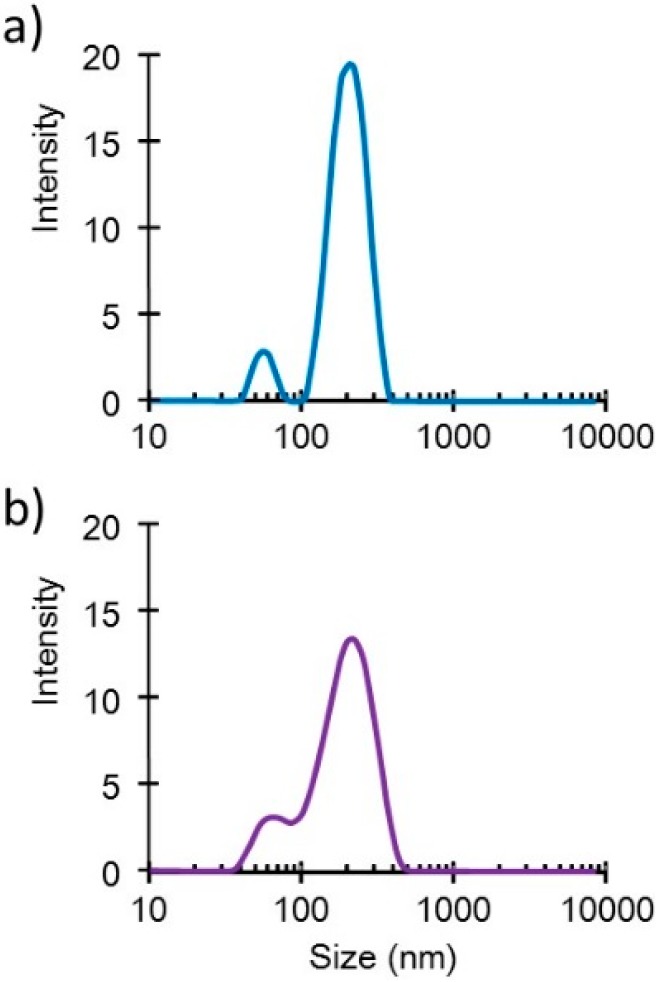
Size distribution profiles of the DNA/(PEI “Max”)/CS complexes (1:12:8) (**a**) before and (**b**) after lyophilization in the presence of 0.1% dextran.

**Figure 2:**
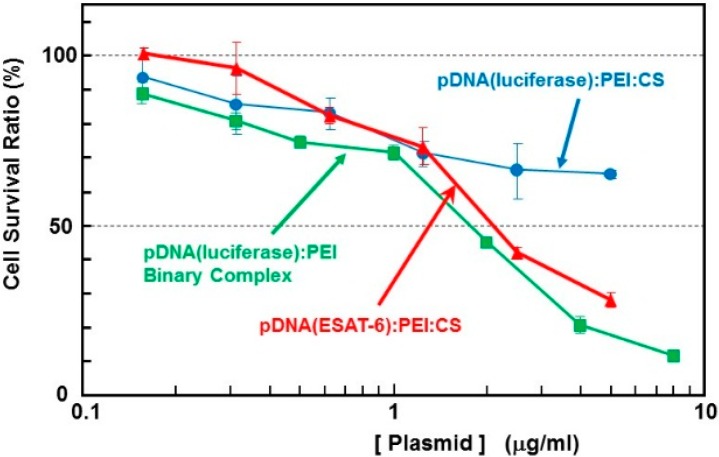
Cytotoxicity of the complexes with plasmid encoding ESAT-6 or luciferase in B16 melanoma cells.

Small DNA complex particles (~200 nm in diameter) were obtained through the freeze-drying method (Figure 1). These small plasmid complexes expressed high *in vivo* transfection efficiency, as reported in another article [2]. The cytotoxicity of the complex containing the *ESAT-6* gene plasmid was estimated using the WST-1 assay. The survival rate in cells treated with binary- or ternary-complexes comprising *luciferase*-encoding pDNA was also simultaneously examined (Figure 2). The pDNA (*luciferase*)/PEI binary complex demonstrated relatively higher cytotoxicity than the pDNA (*luciferase*)/PEI/CS ternary complex. This could be attributed to the surface charge of the complexes. The DNA/PEI binary complex has a positive ζ-potential (~20 mV), while the DNA/PEI/CS ternary complex has a negative ζ-potential (−30 to −40 mV). Nanoparticles with a positive surface charge are known to demonstrate higher cytotoxicity than those with negative charge. The toxicity of the complex expressing ESAT-6, at up to 1 μg/mL [DNA], was lower than that of the *luciferase*-encoding plasmid. However, at concentrations higher than 2 μg/mL [DNA], a relatively higher toxicity on the cells was observed. Cell survival after incubation with the complex at a pDNA (*ESAT-6*) concentration of 50 μg/mL was 28% of the control, while 65% of the cells treated with the same concentration of the pDNA (*luciferase*) complex survived. The mechanism of the relatively higher toxicity of the pDNA (*ESAT-6*) complex is not clear, but might be due to the pore formation [11] or membrane lysing [12] property of the ESAT-6 protein.

**Figure 3:**
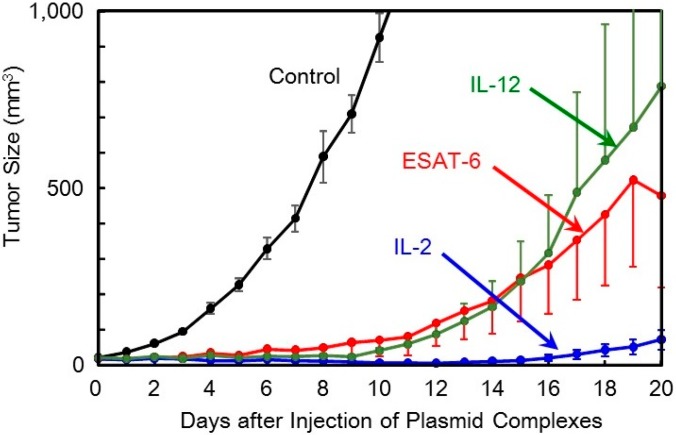
Therapeutic efficacy of the DNA/(PEI “Max”)/CS complexes (1:12:8) comprising plasmid encoding ESAT-6, IL-2, or IL-12 in tumor-bearing mice.

The anti-tumor efficacy of the *ESAT-6* gene was then examined compared to that of the *IL-2* and *IL-12* genes. The ternary complex containing 100 μg of the plasmid encoding ESAT-6, or the cytokines, was intratumorally injected into tumor-bearing mice every other day for 5 treatments. Transfection with the *ESAT-6* gene induced a significant anti-tumor effect, and tumor growth was markedly suppressed compared to the control mice which received nothing. Tumor suppression induced by the *ESAT-6* gene was similar to that observed with the *IL-12* gene (Figure 3). Judging from the average tumor size, IL-2 transfection demonstrated the most significant tumor growth regression, among them. However, tumor recurrence occurred in all the mice treated by the cytokine gene, while complete tumor clearance and prolonged survival over 125 days was observed in 2 out of 10 mice treated with pDNA (*ESAT-6*) (Figure 6).

**Figure 4:**
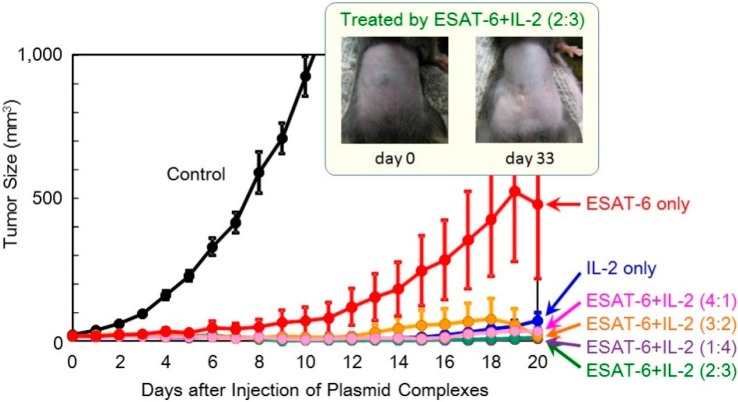
Therapeutic efficacy of combination treatment with DNA complexes expressing ESAT-6 and IL-2 in tumor-bearing mice.

**Figure 5:**
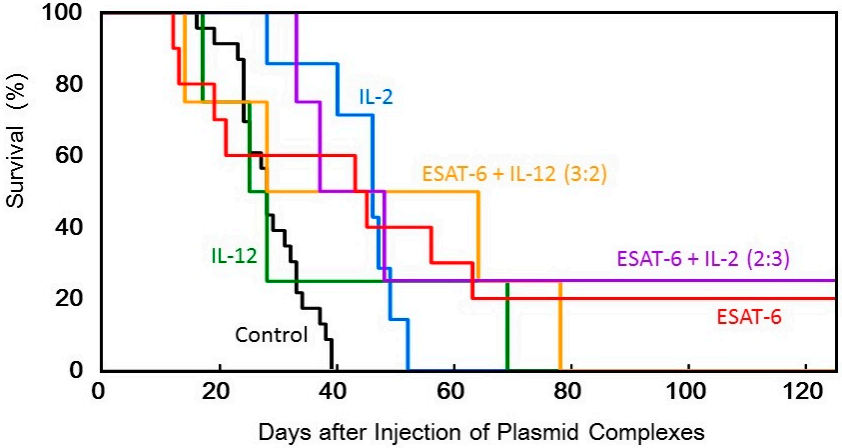
Survival of tumor-bearing mice treated with the transfection of the DNA complexes expressing ESAT-6, IL-2, or IL-12, or co-transfection of the DNA complexes expressing ESAT-6 and the cytokines.

**Figure 6:**
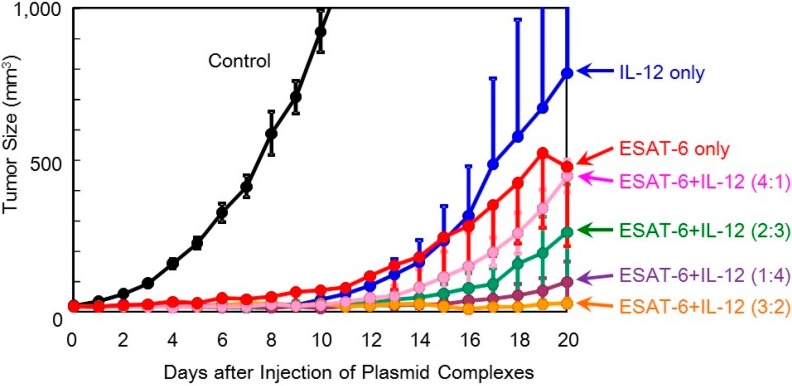
Therapeutic efficacy of co-transfection of the DNA complexes expressing ESAT-6 and IL-12 in tumor-bearing mice.

In order to achieve higher efficacy, co-transfection of ESAT-6 and the IL-2 or IL-12 cytokine was tested. A suspension of the pDNA (*ESAT-6*) complex was mixed with that of pDNA encoding IL-2 or IL-12, and administered to mice. The total amount of the plasmid administered was 100 μg per injection. The superior antitumor efficacy observed using the *IL-2* gene was further improved by the addition of the *ESAT-6* gene (Figure 4). Furthermore, complete regression of the solid tumor was attained in 2 out of 8 mice treated with co-transfection at the mixing ratio of ESAT-6:IL-2 of 2:3 or 3:2, with no recurrence for 125 days (Figure 5). Co-transfection of the pDNA (*ESAT-6*) and pDNA (*IL-12*) complexes also demonstrated a greater antitumor effect than either treatment alone (Figure 6).

**Figure 7:**
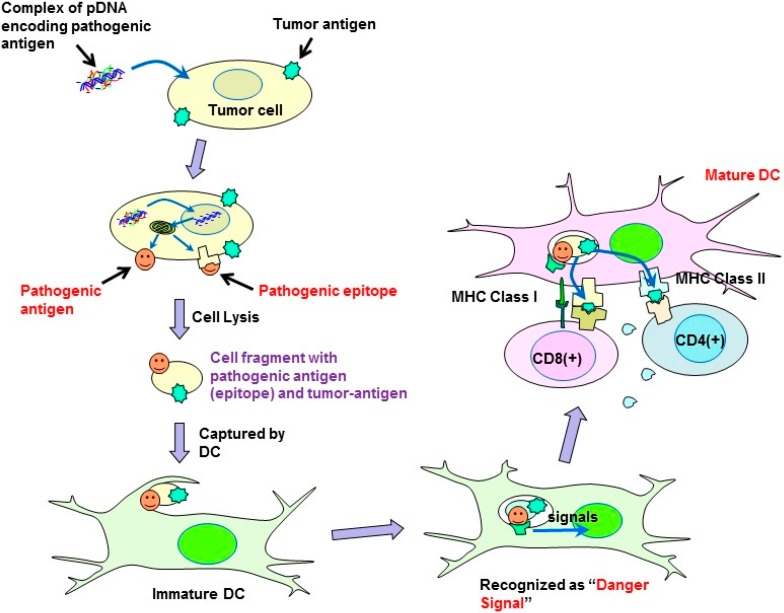
Possible mechanism of the antitumor effect induced by plasmids encoding pathogenic antigens.

In the present study, a syngeneic B16 melanoma tumor model was utilized. In this model, tumor cells might escape from immune surveillance, as the tumor cells are altered cells derived from genetically identical mice. Similarly, in established primary tumors, malignant transformed cells generally display weak immunogenicity, which often limit the efficacy of immunotherapy. Cytokine genes have been used in cancer gene therapy to enhance immunity. However, it is difficult to activate immunity against poorly immunogenic tumor antigens, and, thus, utilizing cytokine-transfection as a monotherapy is not sufficient to prevent tumor escape. To overcome the disadvantage of weak immunogenicity, in this study, the chemically synthesized gene of the pathogenic antigen, ESAT-6, was introduced into tumor cells. The ESAT-6 protein produced in tumor cells may be directly presented on tumor cell surfaces due to the lipid membrane-interacting properties of the protein [10]. Otherwise, the protein can also be degraded in the proteasome into epitopes, which would be transported into the endoplasmic reticulum, and finally presented on the cell surface in the context of the major histocompatibility complex (MHC) class I. In either case, the antigenic structure of ESAT-6 would be exposed to the tumor cell membrane. It would be captured by antigen-presenting cells (APCs) together with tumor-associated antigens (TAAs) existing on the same surface membrane. APCs would recognize the ESAT-6 or its fragment as a “danger signal”, and be expected to mature to cross-prime T cells against ESAT-6 and also TAAs even with weak immunogenicity (Figure 7). Recently, a genetically engineered live tumor cell vaccine that stably expresses glycosyl-phosphatidylinositol-anchored ESAT-6 and IL-21 was developed [14]. It could effectively induce tumor suppression in mice bearing the same tumor from which the vaccine cell was derived. The antitumor efficacy of the viable cell vaccine could be attributed to its ability to produce both a “danger signal” and TAAs. In order to express TAAs, which is necessary for effective anti-tumor immunity, the cell vaccine should be generated from the tumor cell collected from the patient. Depending on the properties of the tumor cells, the process may be time consuming and difficult. Transforming the tumor cells by *in vivo* transfection with “danger signal”-inducing genes, as described in the present study, is a simple method to produce a tumor cell simultaneously expressing both “danger signals” and TAAs.

Several types of genes coding TAAs have been identified, and used in the design of DNA cancer vaccines. The results from clinical trials demonstrate that they are safe and have several advantages. However, much improvement is still required to make them sufficiently effective in the clinic. A number of TAAs are also expressed on normal tissues, albeit at different quantities. Thus, inducing immunity against target TAAs could also lead to adverse autoimmunity. Moreover, TAAs are often weakly immunogenic and the immune repertoire in patients may have already been tolerized to these TAAs. Additional strategies are, thus, required to activate effective immunity against poorly immunogenic tumor antigens.

The strategy for overcoming the weak immunogenicity of natural tumor antigens, as presented in this study, is to transform the tumor cells by transfection of “danger signals”. This strategy introduces a highly immunogenic “synthetic tumor antigen” on tumor cell surfaces to activate the immune system. For this purpose, any heterologous protein may possibly serve as the “danger signal”. However, in our preliminary experiments, luciferase-transfection induced little to no tumor growth suppression. High immunogenicity and other characteristics of ESAT-6 protein facilitate a favorable anti-tumor immune response. The mechanism of immune stimulation by ESAT-6 is now under investigation, together with other pathogenic antigens. This antitumor strategy could theoretically be effective in several different types of tumors, and is expected to open up a new way to overcome the current obstacles in cancer immunotherapy, including T cell tolerance against tumor antigens to evoke effective antitumor immune response.

## 4. Conclusions

Intratumoral administration of *ESAT-6* gene, a pathogenic antigen from *Mycobacterium tuberculosis* (TB), demonstrated highly effective tumor growth suppression in syngeneic tumor-bearing mice. Co-transfection of cytokines with ESAT-6 significantly enhanced the antitumor effect of ESAT-6. The TB antigen presented on the tumor cell surfaces would be recognized by APCs as a “danger signal”, leading to the activation of an antitumor immune response. This cancer immunotherapeutic strategy is expected to break the tolerance against TAAs with weak immunogenicity and block tumor mediated immune escape.
